# External validation of the D-PACE score for predicting late high-grade atrioventricular block after transcatheter aortic valve replacement in a Colombian cohort

**DOI:** 10.3389/fcvm.2026.1905818

**Published:** 2026-10-14

**Authors:** Juan David Zuluaga-Monroy, Hugo Oswaldo Alvarado Mora, Diana Fernanda Bohórquez González, Joshua Kock Sierra, Edgar Fernando Hurtado Ordoñez

**Affiliations:** 1 Department of Interventional Cardiology, Fundación Abood Shaio, Bogotá, Colombia; 2 Department of Clinical Epidemiology, Universidad de La Sabana, Chía, Colombia

**Keywords:** atrioventricular block, conduction disturbances, D-PACE score, external validation, Latin America, pacemaker implantation, risk stratification, transcatheter aortic valve replacement

## Abstract

**Background:**

Transcatheter aortic valve replacement (TAVR) carries a clinically significant risk of late high-grade atrioventricular block (AVB), yet no validated tool exists to predict this complication in Latin-American populations. The D-PACE score was developed in European cohorts for this purpose.

**Methods:**

In a retrospective cohort of 175 consecutive TAVR procedures at a Colombian center (2012–2025), 141 patients met inclusion criteria for D-PACE validation. The primary outcome was late high-grade AVB (≥Mobitz type II) occurring between 24 h and 30 days post-TAVR. Discrimination, logistic-regression calibration, and decision curve analysis were performed.

**Results:**

Late AVB occurred in 31 patients (22.0%), with 80.6% of events within 72 h. The D-PACE score demonstrated good discrimination (AUC 0.82; 95% CI 0.71–0.92; sensitivity 77.4%, specificity 86.4% at a cutoff of 5.5 points; NPV 93.1%). Late AVB rates rose progressively across strata: 5.9% (low-risk, score 0–3), 8.8% (intermediate, 4–5), and 61.5% (high-risk, ≥6), with the high-risk group concentrating 77.4% of all events. Decision curve analysis confirmed net clinical benefit across threshold probabilities from 8% to 60%. All four low-risk events occurred among valve-in-valve patients assigned a score of 0 per the original algorithm; excluding this subgroup (*N* = 114), the AUC rose to 0.91 (95% CI 0.85–0.96) with zero low-risk events. In an exploratory analysis applying the full seven-variable scoring uniformly, including valve-in-valve cases, the AUC was 0.90 (95% CI 0.85–0.95; NPV 96.7%), and 0.91 (95% CI 0.78–1.00) within the valve-in-valve subgroup (*N* = 27, 4 events).

**Conclusions:**

Applied as originally specified, the D-PACE score showed good discrimination for late high-grade AVB in this cohort (AUC 0.82; 95% CI 0.71–0.92). The algorithm's automatic low-risk classification of valve-in-valve patients was not supported (14.8% AVB rate). Discrimination was higher both in the sensitivity analysis excluding valve-in-valve cases and in the exploratory analysis applying the seven-variable scoring uniformly, indicating that the scoring variables transfer well while the valve-in-valve exemption warrants reconsideration. These findings provide the first external validation of D-PACE in Latin America.

## 1 Introduction

Aortic stenosis (AS) is the most prevalent primary valvular heart disease worldwide, affecting an estimated 12.6 million individuals and accounting for 41.2% of all primary valvular pathologies (1). Transcatheter aortic valve replacement (TAVR) has evolved from a last-resort option for inoperable patients to the preferred treatment for older adults with severe AS across all surgical risk categories, consistently surpassing surgical aortic valve replacement in procedural volume (2–4).

Despite its transformative impact, TAVR carries a non-negligible risk of conduction disturbances owing to the anatomical proximity of the atrioventricular conduction system to the aortic annulus. Mechanical compression by the prosthesis and calcium displacement can injure the bundle of His and its branches—a process recently delineated through intracardiac electrophysiology mapping and high-resolution imaging (5)—resulting in new-onset left bundle branch block (LBBB) in approximately 20% and high-grade atrioventricular block (AVB) requiring permanent pacemaker implantation (PPI) in 10%–25% of patients, depending on the valve platform and implant technique (6–9). These conduction disturbances are not benign: they are independently associated with prolonged hospitalization, higher readmission rates, left ventricular dyssynchrony, and impaired long-term survival (6, 7, 10, 11).

Although most high-grade AVBs manifest within the first 24 h, delayed events occurring between 24 h and 30 days after the procedure account for a clinically meaningful subset and remain the primary driver of prolonged post-procedural monitoring (12, 13). The prospective CONDUCT-TAVI study (14) reported that late PPI (>48 h) accounts for 7.5% of the overall 21.0% PPI rate, underscoring the continued relevance of delayed conduction events. The unpredictability of these late events has hindered the adoption of early-discharge protocols; observational data indicate that only 12% of TAVR patients are discharged within 24 h and 31% within 48 h (15).

Several risk-prediction tools have been proposed to address this gap, including the two-step pre- and post-procedural risk score by Janiszewski et al. (16) and the CONDUCT-TAVI score (14). More recently, Bendandi et al. (17) developed the D-PACE score (Delayed atrioventricular block Prediction for eArly disChargE), a practical risk-stratification tool that combines seven weighted variables: self-expanding valve use (1 point), pre-existing RBBB (2 points), new-onset persistent LBBB (3 points), new-onset persistent RBBB (4 points), implantation depth (0–4 points), preprocedural PR interval duration (0–3 points), and next-day PR interval increase (0–3 points). In a multicenter European study (derivation *n* = 915, validation *n* = 936), the score achieved an area under the curve (AUC) of 0.879 in the derivation set and 0.799 in external validation, stratifying patients into low-risk (score 0–3; predicted AVB <2%), intermediate-risk (score 4–5; 2%–5%), and high-risk (score ≥6; ≥5%) categories for late AVB.

However, the performance of any predictive model may vary across populations with different genetic backgrounds, anatomical characteristics, device-use patterns, and institutional practices. Latin-American TAVR registries remain underrepresented in the literature, and no validation of D-PACE has been reported in this region. Because the distribution of key risk factors including the prevalence of self-expanding valve use, implant depth practices, and baseline conduction abnormalities may differ substantially from European cohorts, local validation is essential before clinical adoption.

The aim of this study was to externally validate the D-PACE score in a consecutive Colombian TAVR cohort and to characterize the clinical, procedural, and electrophysiological profile of patients who develop late high-grade AVB.

## 2 Materials and methods

### 2.1 Study design and population

This was a single-center, retrospective cohort study conducted at Fundación Abood Shaio, a tertiary cardiovascular center in Bogotá, Colombia. The study is reported in accordance with the Strengthening the Reporting of Observational Studies in Epidemiology (STROBE) guidelines and the Transparent Reporting of a multivariable prediction model for Individual Prognosis or Diagnosis (TRIPOD) statement for external validation studies (18); the completed checklists are provided as Supplementary Material. All consecutive patients aged ≥18 years who underwent TAVR for severe AS between January 1, 2012, and March 31, 2025, were screened. The study was approved by the institutional ethics committee, and informed consent was waived due to the retrospective design.

### 2.2 Inclusion and exclusion criteria

**Inclusion criteria:**

- Age ≥18 years with severe AS confirmed by echocardiography
- TAVR performed during the study period as determined by the institutional Heart Team

**Exclusion criteria:**

- Prior permanent pacemaker or pre-existing indication for cardiac stimulation device
- Permanent or persistent atrial fibrillation on the preprocedural ECG, precluding PR interval measurement required for D-PACE calculation
- Cardiac stimulation device implanted within 30 days post-TAVR for an indication other than high-grade AVB
- High-grade AVB occurring within the first 24 h post-TAVR
- Incomplete clinical or electrocardiographic follow-up data
- Death within 30 days without prior development of high-grade AVB
- Transcatheter valve implanted in the mitral position or valve-in-valve in a previously dysfunctional mitral prosthesis

### 2.3 Data collection

Data were extracted from electronic health records, catheterization laboratory databases, operative reports, and electrocardiographic registries. Variables collected included demographics (age, sex, body mass index, body surface area), comorbidities (hypertension, dyslipidemia, diabetes mellitus, heart failure, chronic obstructive pulmonary disease, chronic kidney disease, atrial fibrillation, coronary artery disease, prior cardiac surgery), Society of Thoracic Surgeons Predicted Risk of Mortality (STS-PROM), echocardiographic parameters (aortic valve area, gradients, left ventricular ejection fraction), computed tomography angiography findings (bicuspid morphology), and procedural details (valve type, model, size, implant depth, pre/post-dilation, paravalvular leak).

Electrocardiographic data were obtained from three time points, following the protocol established by Bendandi (17): (1) a preprocedural ECG recorded within 72 h before TAVR, (2) a postprocedural ECG recorded within 4 h after valve deployment, and (3) a next-day ECG recorded between 12 and 24 h post-TAVR. All ECGs were 12-lead recordings reviewed for rhythm, heart rate, PR interval, QRS duration, and conduction abnormalities. Conduction disturbances were defined according to the AHA/ACCF/HRS consensus document on ECG interpretation (19): first-degree AVB (PR >200 ms), LBBB and RBBB per standard criteria, and high-grade AVB (third-degree, second-degree Mobitz type II, 2:1, or advanced block). New-onset bundle branch blocks were classified as persistent (present in both postprocedural and next-day ECGs) or transient (present in the postprocedural ECG but resolved in the next-day ECG), consistent with the original D-PACE definitions.

### 2.4 D-PACE score calculation

The D-PACE score was calculated for each patient according to the original publication by Bendandi et al.(17), incorporating seven weighted variables: (1) self-expanding valve use (1 point), (2) pre-existing RBBB (2 points), (3) new-onset persistent LBBB (3 points), (4) new-onset persistent RBBB (4 points), (5) angiographic implantation depth below the aortic annulus (<3 mm = 0, 3–4.9 mm = 1, 5–6.9 mm = 2, ≥7 mm = 4 points), (6) preprocedural PR interval duration (<150 ms = 0, 150–199 ms = 1, 200–249 ms = 2, ≥250 ms = 3 points), and (7) next-day PR interval increase compared to baseline (<1 ms = 0, 1–19 ms = 1, ≥20 ms = 3 points). The total score is the sum of these seven components and ranges from 0 to 14 points. Implantation depth was measured from angiographic images as the distance from the lowest point of the non-coronary cusp to the lower edge of the valve stent in a cusp-overlap or three-cusp projection; measurements were performed by two experienced interventional cardiologists, with disagreements resolved by consensus. Independent duplicate measurements were not obtained, and inter-observer agreement for this variable could therefore not be quantified. Patients with valve-in-valve procedures on conventional surgical prostheses were not excluded from the cohort. In the primary analysis they were assigned a D-PACE score of 0 and analyzed within the full cohort, exactly as the published algorithm specifies and consistent with the original study's finding that this subgroup had negligible late AVB risk; they were additionally examined as a separate subgroup in the sensitivity and exploratory analyses described below. Patients were stratified into three risk categories: low (0–3 points), intermediate (4–5 points), and high (≥6 points). All D-PACE scores were independently calculated by two investigators (JDZ and JHK); discrepancies were resolved by consensus review with a third investigator (EFH), with the final adjudicated scores used for all analyses.

### 2.5 Outcomes

The primary outcome was late high-grade AVB, defined as the occurrence of second-degree Mobitz type II, 2:1, advanced, or third-degree AVB between 24 h and 30 days post-TAVR, confirmed by ECG or continuous telemetry monitoring. All patients underwent continuous in-hospital telemetry for a minimum of 72 h after valve deployment, with 12-lead electrocardiography at discharge. Follow-up to 30 days was complete for the entire cohort and combined three sources: a scheduled outpatient visit with 12-lead electrocardiography, telephone contact with the patient or a family member to ascertain survival and any pacemaker implantation, and review of institutional records for readmissions and for pacemaker procedures performed at our center. Ambulatory rhythm monitoring was not part of the follow-up protocol. No event meeting the electrocardiographic definition was excluded on the basis of symptom status.

Secondary outcomes included: new-onset LBBB, new first-degree AVB, PPI within 30 days, all-cause 30-day mortality, cardiovascular mortality, and length of hospital stay.

### 2.6 Statistical analysis

Continuous variables are presented as mean ± standard deviation (SD) or median [interquartile range (IQR)], depending on the distribution assessed by the Shapiro–Wilk test. Categorical variables are expressed as absolute frequencies and percentages.

**Discrimination** was evaluated by constructing a ROC curve for the D-PACE score against the primary outcome, with the AUC and its 95% confidence interval (CI) calculated using the stratified percentile bootstrap method with 20,000 resamples (random seed 2026); in the subgroup analyses, the direction of the ROC comparison was fixed *a priori*. Sensitivity, specificity, positive predictive value (PPV), and negative predictive value (NPV) were determined at the optimal cutoff identified by the Youden index.

**Calibration** was assessed at two levels. First, because the original D-PACE algorithm assigns patients to risk strata rather than providing individual predicted probabilities, observed event rates were compared with the stratum definitions from the derivation study. Second, a local logistic regression model was fitted with AVB occurrence as the dependent variable and the raw D-PACE score as a single continuous predictor, yielding an intercept, slope, odds ratio per point, and Brier score; this model represents a site-specific recalibration rather than a direct test of the original algorithm's predictions.

**Clinical utility** was evaluated by decision curve analysis (DCA), which quantifies the net benefit of model-guided decisions compared with default strategies of treating all or treating no patients across a range of clinically relevant threshold probabilities (20). Optimism correction was applied using 1,000 bootstrap resamples to ensure that clinical utility estimates were not inflated by overfitting.

A planned sensitivity analysis was performed after removing the 27 valve-in-valve patients, who were classified as low-risk in the primary analysis per the original algorithm, to evaluate D-PACE performance based exclusively on the seven-variable scoring in the remaining cohort (17). An additional exploratory *post hoc* analysis applied the full seven-variable D-PACE scoring to the valve-in-valve subgroup (i.e., overriding the automatic low-risk classification) to determine whether the scoring variables could discriminate late AVB risk within this subgroup; given the small sample size (*N* = 27, 4 events), the results of this analysis should be regarded as hypothesis-generating.

Stratum-specific event rates are reported with exact binomial (Clopper–Pearson) 95% confidence intervals.

All statistical analyses were performed using R version 4.2.2 (R Foundation for Statistical Computing, Vienna, Austria) with packages from the tidyverse ecosystem (v2.0.0), rms (v7.0.0), pROC (v1.18.5) for ROC analysis and bootstrap AUC confidence intervals, dcurves (v0.4.0) for decision curve analysis, and ggplot2 (v3.5.1). The analysis environment was controlled with renv (v1.1.2) for reproducibility.

## 3 Results

### 3.1 Study population

Between January 2012 and March 2025, 175 TAVR procedures were performed. After applying exclusion criteria, 141 patients were included in the D-PACE validation cohort (Figure 1). Of these, 31 (22.0%) developed late high-grade AVB and 110 (78.0%) did not.

**Figure 1:**
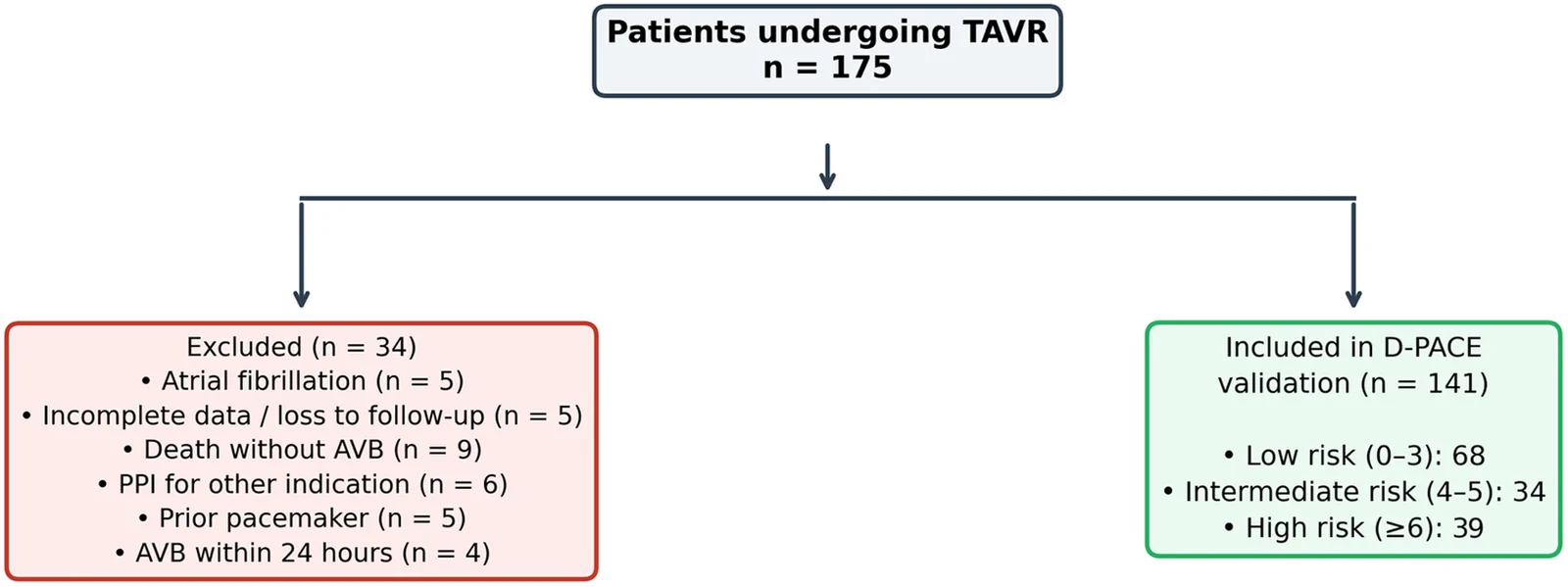
Flowchart of patient selection for D-PACE validation. Of 175 consecutive TAVR procedures performed between January 2012 and March 2025, 34 patients were excluded: atrial fibrillation precluding PR interval measurement (*n* = 5), incomplete data or loss to follow-up (*n* = 5), death within 30 days without AVB (*n* = 9), prior permanent pacemaker (*n* = 5), pacemaker implantation for indication other than high-grade AVB (*n* = 6), and high-grade AVB within the first 24 h (*n* = 4). No patients were excluded for mitral-position implantation. The final analytic cohort comprised 141 patients

### 3.2 Baseline characteristics

The baseline characteristics of the study population are summarized in Table 1. Mean age was 79.6 ± 6.0 years, with 72 patients (51.1%) being female. Predominant comorbidities included hypertension (87.9%), dyslipidemia (65.2%), heart failure (45.4%), coronary artery disease (39.0%), and diabetes mellitus (29.1%). The mean STS-PROM was 6.0 ± 3.6%. Prior cardiac surgery had been performed in 41 patients (29.1%); of these, 27 (19.1%) had a prior surgical aortic bioprosthesis and underwent valve-in-valve TAVR.

**Table 1:** Baseline demographic and clinical characteristics.

Variable	Total (*N* = 141)	Late AVB (*N* = 31)	No AVB (*N* = 110)
Age (years), mea*n* ± SD	79.6 ± 6.0	81.3 ± 5.3	79.2 ± 6.1
Female sex, *n* (%)	72 (51.1)	18 (58.1)	54 (49.1)
BMI (kg/m^2^), mean ± SD	25.5 ± 4.4	25.0 ± 3.9	25.6 ± 4.5
BSA (m^2^), mean ± SD	1.7 ± 0.2	1.7 ± 0.2	1.7 ± 0.2
STS-PROM (%), mean ± SD	6.0 ± 3.6	6.9 ± 4.1	5.8 ± 3.4
Hypertension, *n* (%)	124 (87.9)	28 (90.3)	96 (87.3)
Dyslipidemia, *n* (%)	92 (65.2)	17 (54.8)	75 (68.2)
Diabetes mellitus, *n* (%)	41 (29.1)	8 (25.8)	33 (30.0)
Heart failure, *n* (%)	64 (45.4)	15 (48.4)	49 (44.5)
Smoking history (current/former), *n* (%)	71 (50.4)	19 (61.3)	52 (47.3)
COPD, *n* (%)	21 (14.9)	5 (16.1)	16 (14.5)
Chronic kidney disease (GFR <60), *n* (%)	34 (24.1)	5 (16.1)	29 (26.4)
History of atrial fibrillation, *n* (%)	18 (12.8)	6 (19.4)	12 (10.9)
Coronary artery disease, *n* (%)	55 (39.0)	12 (38.7)	43 (39.1)
Prior PCI, *n* (%)	42 (29.8)	8 (25.8)	34 (30.9)
Prior cardiac surgery, *n* (%)	41 (29.1)	6 (19.4)	35 (31.8)
LVEF (%), mean ± SD	52.3 ± 13.5	52.2 ± 12.6	52.3 ± 13.8
LVEF <40%, *n* (%)	26 (18.4)	4 (12.9)	22 (20.0)
NYHA functional class III–IV, *n* (%)	89 (63.1)	19 (61.3)	70 (63.6)

Between-group comparisons are descriptive; the study was not powered for inferential testing of baseline differences. AVB, atrioventricular block; BMI, body mass index; BSA, body surface area; COPD, chronic obstructive pulmonary disease; GFR, glomerular filtration rate; LVEF, left ventricular ejection fraction; NYHA, New York Heart Association; PCI, percutaneous coronary intervention; STS-PROM, Society of Thoracic Surgeons Predicted Risk of Mortality.

Patients who developed late AVB were slightly older (81.3 ± 5.3 vs. 79.2 ± 6.1 years), more frequently female (58.1% vs. 49.1%), and had a higher prevalence of prior smoking history (61.3% vs. 47.3%) and history of atrial fibrillation (19.4% vs. 10.9%) compared to those without AVB, although prior cardiac surgery was less common in the AVB group (19.4% vs. 31.8%).

### 3.3 Echocardiographic and CT angiography findings

Mean aortic valve area was 0.7 ± 0.2 cm^2^, indexed aortic valve area 0.4 ± 0.2 cm^2^/m^2^, mean echocardiographic gradient 45.9 ± 15.8 mmHg, and peak velocity 4.2 ± 0.7 m/s, without meaningful differences between groups. Mean LVEF was 52.3 ± 13.5%. CT angiography was performed in 97.2% of patients; bicuspid valve morphology was present in 31 (22.0%), predominantly type 1A (12.8%).

### 3.4 Procedural characteristics

Procedural details are presented in Table 2. All procedures were performed via the transfemoral approach. Self-expanding valves were implanted in 81 patients (57.4%) and balloon-expandable valves in 60 (42.6%). The most frequently used models were SAPIEN 3 (40.4%), CoreValve (19.9%), Acurate Neo2 (9.9%), and Evolut R (9.9%). Mean implant depth was 4.7 ± 2.4 mm overall, notably deeper in the AVB group (6.2 ± 2.7 vs. 4.3 ± 2.2 mm). Valve-in-valve procedures accounted for 19.1% of cases.

**Table 2:** Procedural characteristics.

Variable	Total (*N* = 141)	Late AVB (*N* = 31)	No AVB (*N* = 110)
Valve-in-valve, *n* (%)	27 (19.1)	4 (12.9)	23 (20.9)
Valve type			
Self-expanding, *n* (%)	81 (57.4)	22 (71.0)	59 (53.6)
Balloon-expandable, *n* (%)	60 (42.6)	9 (29.0)	51 (46.4)
Most common models			
SAPIEN 3, *n* (%)	57 (40.4)	9 (29.0)	48 (43.6)
CoreValve, *n* (%)	28 (19.9)	9 (29.0)	19 (17.3)
Evolut R, *n* (%)	14 (9.9)	5 (16.1)	9 (8.2)
Acurate Neo2, *n* (%)	14 (9.9)	4 (12.9)	10 (9.1)
Portico, *n* (%)	6 (4.3)	2 (6.5)	4 (3.6)
Other, *n* (%)	22 (15.6)	2 (6.5)	20 (18.2)
Implant depth (mm), mean ± SD	4.7 ± 2.4	6.2 ± 2.7	4.3 ± 2.2
Pre-dilation, *n* (%)	78 (55.3)	18 (58.1)	60 (54.5)
Post-dilation, *n* (%)	36 (25.5)	5 (16.1)	31 (28.2)
Significant paravalvular leak (≥moderate), *n* (%)	4 (2.8)	0 (0.0)	4 (3.6)
Technical success, *n* (%)	126 (89.4)	25 (80.6)	101 (91.8)

Technical success was defined according to the Valve Academic Research Consortium–3 (VARC-3) criteria: correct positioning of a single prosthetic valve, intended performance without moderate or severe prosthetic valve regurgitation, and no procedural mortality (34). AVB, atrioventricular block.

Self-expanding valve use was more prevalent in patients who developed late AVB (71.0% vs. 53.6%). Pre-dilation was performed in 55.3% and post-dilation in 25.5%, without substantial differences between groups. Significant (≥moderate) paravalvular leak occurred in 2.8%.

### 3.5 Electrocardiographic findings

Pre-procedural and 24-hour post-procedural ECG characteristics are detailed in Table 3. All 141 included patients were in sinus rhythm on the preprocedural ECG; patients with a history of atrial fibrillation listed in Table 1 had paroxysmal episodes but were in sinus rhythm at the time of recording. Baseline PR interval was 165.6 ± 30.8 ms and QRS duration 103.9 ± 19.9 ms. Pre-existing first-degree AVB was present in 14.9%, LBBB in 3.5%, and RBBB in 9.2%. Patients in the AVB group had a higher prevalence of baseline RBBB (19.4% vs. 6.4%).

**Table 3:** Electrocardiographic characteristics pre- and post-TAVR.

Variable	Total (*N* = 141)	Late AVB (*N* = 31)	No AVB (*N* = 110)
Baseline ECG			
PR interval (ms), mean ± SD	165.6 ± 30.8	169.9 ± 27.8	164.3 ± 31.6
QRS duration (ms), mean ± SD	103.9 ± 19.9	109.8 ± 21.7	102.2 ± 19.2
First-degree AVB, *n* (%)	21 (14.9)	5 (16.1)	16 (14.5)
LBBB, *n* (%)	5 (3.5)	2 (6.5)	3 (2.7)
RBBB, *n* (%)	13 (9.2)	6 (19.4)	7 (6.4)
Next-day ECG (12–24 h post-TAVR)			
PR interval (ms), mean ± SD	181.4 ± 41.8	210.0 ± 55.0	173.3 ± 33.3
QRS duration (ms), mean ± SD	117.8 ± 25.0	140.2 ± 18.4	111.5 ± 23.0
Delta PR vs. baseline (ms), median [IQR]	4.0 [0.0–22.0]	20.0 [7.5–48.5]	2.0 [0.0–13.5]
Delta QRS vs. baseline (ms), median [IQR]	6.0 [2.0–22.0]	33.0 [9.0–50.0]	4.0 [0.0–12.8]
New-onset conduction disturbances			
New first-degree AVB, *n* (%)	19 (13.5)	9 (29.0)	10 (9.1)
New persistent LBBB, *n* (%)	40 (28.4)	18 (58.1)	22 (20.0)
Transient LBBB, *n* (%)	5 (3.5)	0 (0.0)	5 (4.5)
New persistent RBBB, *n* (%)	3 (2.1)	2 (6.5)	1 (0.9)
Late high-grade AVB, persistent on 12-lead ECG, *n* (%)	24 (17.0)	24 (77.4)	0 (0.0)
Late high-grade AVB, transient on telemetry, *n* (%)	7 (5.0)	7 (22.6)	0 (0.0)

New-onset bundle branch blocks were classified as persistent (present in both postprocedural and next-day ECGs) or transient (present in postprocedural ECG only, resolved by next-day recording). Five patients had transient LBBB, all in the no-AVB group, with no subsequent conduction events. AVB, atrioventricular block; LBBB, left bundle branch block; RBBB, right bundle branch block.

At 24 h post-TAVR, PR interval increase was markedly greater in the AVB group [median 20.0 ms (IQR 7.5–48.5)] than in the no-AVB group [2.0 ms (IQR 0.0–13.5)], as was QRS widening [33.0 ms [IQR 9.0–50.0] vs. 4.0 ms [IQR 0.0–12.8]]. New-onset LBBB occurred in 28.4% overall (58.1% in the AVB group vs. 20.0% in the no-AVB group), making it the most frequent post-procedural conduction disturbance. An additional 5 patients developed transient LBBB on the postprocedural ECG that had resolved by the next-day recording; none of these patients developed late AVB. New-onset persistent RBBB was observed in 3 patients (2.1%), two of whom developed late AVB.

### 3.6 Conduction disturbances and pacemaker implantation

Late high-grade AVB occurred in 31 patients (22.0%), with a median onset at post-procedural day 2 [IQR 2.0–3.0] (Figure 2), where day 1 denotes the 24–48-hour interval after valve deployment. Most events (25/31, 80.6%) occurred within the first 72 h (days 1–3). Third-degree AVB was the predominant type (28/31, 90.3%), with second-degree Mobitz type II or 2:1 block comprising the remainder (3/31, 9.7%). Of the 31 late high-grade AVB events, 24 (77.4%) were persistent at the time of assessment and documented on a formal 12-lead electrocardiogram, while 7 (22.6%) were transient or paroxysmal episodes captured on continuous telemetry.

**Figure 2:**
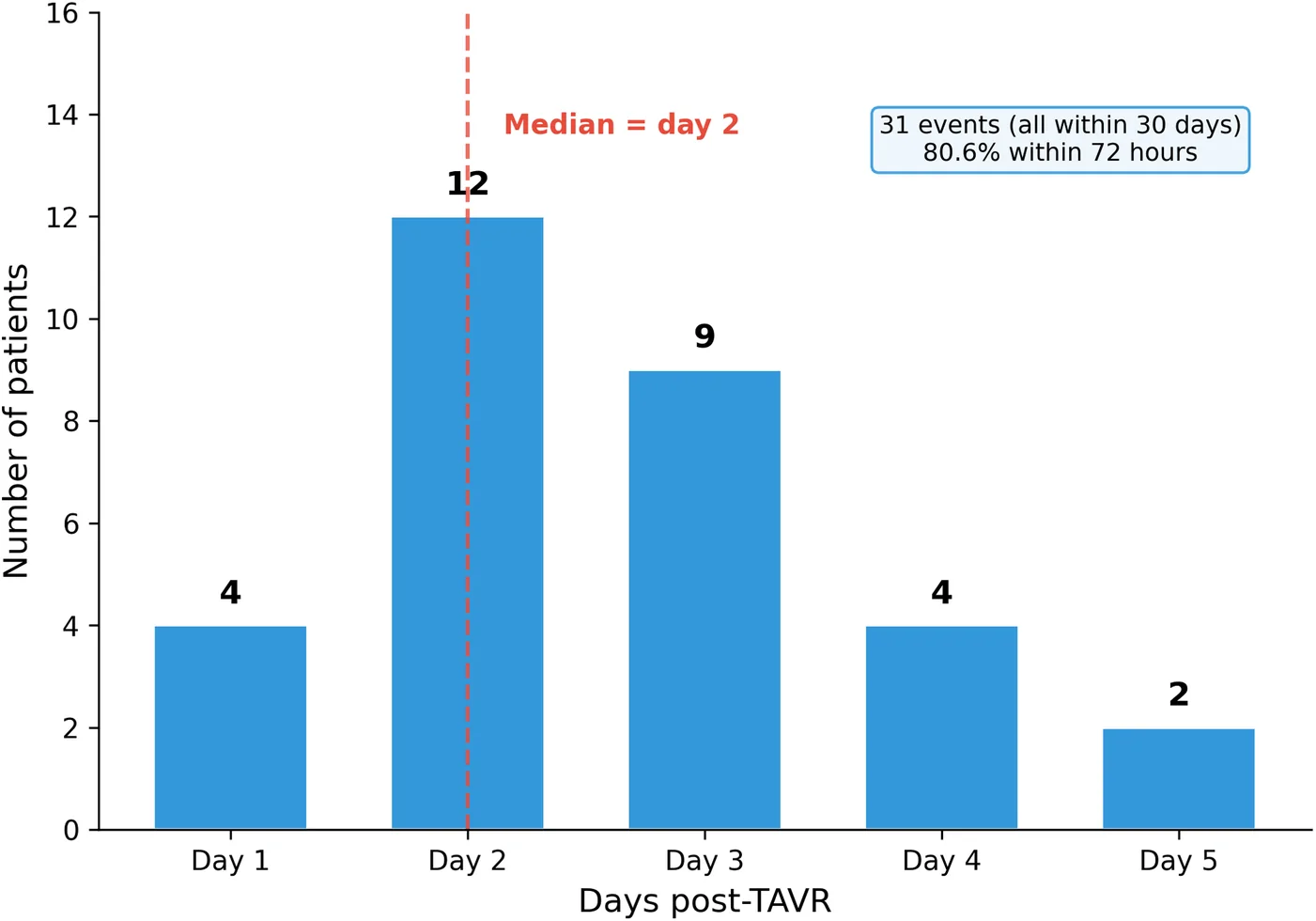
Temporal distribution of late high-grade atrioventricular block onset after TAVR. The bar chart shows the number of patients developing late AVB on each post-procedural day (days 1–5), where day 1 represents the 24–48-hour interval after valve deployment. The majority of events occurred within the first three post–procedural days (days 1–3), with a median onset at day 2.

PPI was performed in 30 of 31 patients who developed late high-grade AVB; one patient died before device implantation. The predominant device type was a dual-chamber pacemaker (27/30, 90.0%); two patients received a single-chamber device and one a cardiac resynchronization therapy defibrillator (CRT-D). Median time to PPI was 5.0 days [IQR 3.0–7.0] post-TAVR.

### 3.7 Outcomes at 30 days

Thirty-day outcomes are summarized in Table 4. Thirty-day all-cause mortality was 3.5% (5/141), with a markedly higher rate in the AVB group (12.9% vs. 0.9%); cardiovascular mortality accounted for 3 of these 5 deaths (all in the AVB group). Mean post-TAVR stay was 7.2 ± 5.6 days (10.4 ± 5.9 days in the AVB group vs. 6.3 ± 5.2 days in the no-AVB group).

**Table 4:** Pacemaker implantation and 30-day outcomes.

Variable	Total (*N* = 141)	Late AVB (*N* = 31)	No AVB (*N* = 110)
PPI implanted, *n* (%)	30 (21.3)	30 (96.8)	0 (0.0)
Time to PPI (days), median [IQR]	5.0 [3.0–7.0]	5.0 [3.0–7.0]	—
Device type: dual-chamber, *n* (%)	27/30 (90.0)	27/30 (90.0)	—
30-day all-cause mortality, *n* (%)	5 (3.5)	4 (12.9)	1 (0.9)
Cardiovascular mortality, *n* (%)	3 (2.1)	3 (9.7)	0 (0.0)
Post-TAVR stay (days), mean ± SD	7.2 ± 5.6	10.4 ± 5.9	6.3 ± 5.2

AVB, atrioventricular block; PPI, permanent pacemaker implantation. Rates are computed within the analytic cohort (*N* = 141), which by construction excludes patients with a prior pacemaker, patients paced within 30 days for an indication other than high-grade AVB, and patients whose high-grade AVB occurred within the first 24 h; the corresponding rates for the whole screened population are given in Section 4.3.

### 3.8 D-PACE score validation

#### 3.8.1 Risk stratification

All 141 patients in the analytic cohort, including the 31 with late high-grade AVB, had complete data for the seven D-PACE variables. The distribution of D-PACE scores across the cohort is shown in Figure 3.

**Figure 3:**
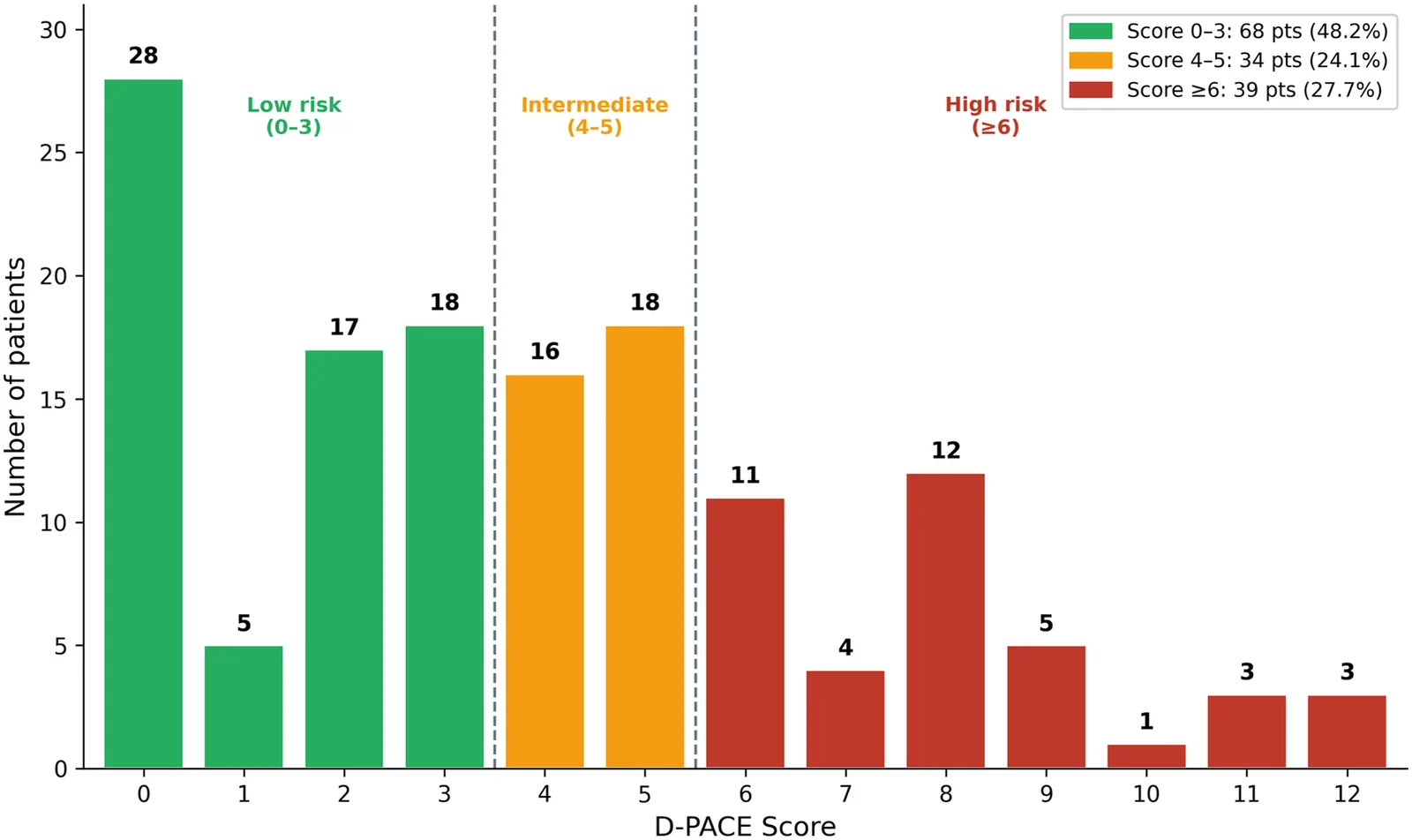
Distribution of D-PACE scores across the study cohort (*N* = 141) in the primary analysis, in which the 27 valve-in-valve patients are assigned a score of 0 per the original algorithm and every other patient receives the sum of their seven components. The histogram displays the frequency of each score value (0–12 points), and the vertical dashed lines indicate the risk-category thresholds at 4 and 6 points. The resulting strata comprise 68 low-risk (48.2%), 34 intermediate-risk (24.1%) and 39 high-risk patients (27.7%); see Table 5.

**Table 5:** D-PACE risk stratification and late AVB rates.

D-PACE stratum	Score range	*N* (%)	Late AVB, *n* (%)	95% CI	% of total events
Low risk	0–3	68 (48.2)	4 (5.9)	1.6–14.4	12.9
Intermediate risk	4–5	34 (24.1)	3 (8.8)	1.9–23.7	9.7
High risk	≥6	39 (27.7)	24 (61.5)	44.6–76.6	77.4
Total		141 (100)	31 (22.0)		100

AVB, atrioventricular block.

The distribution of patients across D-PACE risk strata and corresponding late AVB rates are presented in Table 5. Among the 141 patients, 68 (48.2%) were classified as low-risk (including 27 valve-in-valve patients automatically assigned to this category per the original algorithm), 34 (24.1%) as intermediate-risk, and 39 (27.7%) as high-risk. The late AVB rate increased progressively: 5.9% in the low-risk group, 8.8% in the intermediate-risk group, and 61.5% in the high-risk group. The high-risk stratum concentrated 24 of 31 events (77.4%). Notably, all four low-risk events occurred in valve-in-valve patients.

Among the 114 non-valve-in-valve patients for whom the seven-variable score was computed, the mean total D-PACE score was 7.9 ± 2.1 in the AVB group versus 4.0 ± 2.2 in the no-AVB group [median 8.0 [IQR 6.0–9.0] vs. 4.0 [2.0–5.0]]. Component-level analysis revealed that the score differential between groups was primarily driven by three variables: new-onset persistent LBBB, implantation depth, and next-day PR interval increase. Pre-existing RBBB and self-expanding valve use contributed modestly, while preprocedural PR interval showed minimal between-group variation.

#### 3.8.2 Discrimination

The ROC curve for the D-PACE score yielded an AUC of 0.82 (95% CI 0.71–0.92; percentile bootstrap), indicating good discriminative ability (Figure 4; Table 6). The optimal cutoff identified by the Youden index was 5.5 points—coinciding with the boundary between the intermediate-risk (4, 5) and high-risk (≥6) strata defined in the original study—achieving a sensitivity of 77.4%, specificity of 86.4%, positive predictive value (PPV) of 61.5%, and negative predictive value (NPV) of 93.1%.

**Figure 4:**
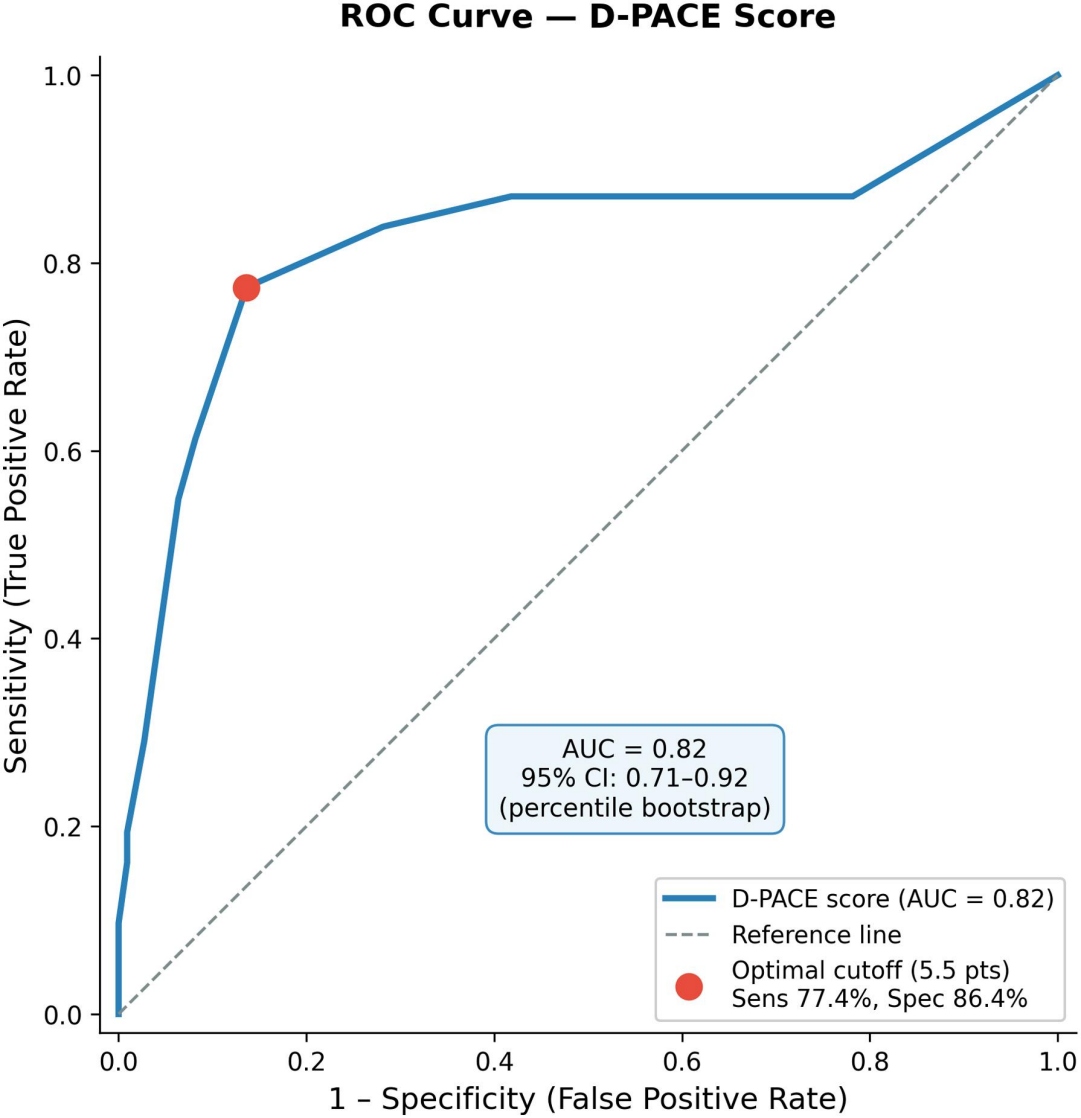
Receiver operating characteristic (ROC) curve for the D-PACE score predicting late high-grade atrioventricular block. AUC = 0.82 (95% CI 0.71–0.92; percentile bootstrap).

**Table 6:** Discrimination and calibration performance of the D-PACE score.

Metric	Value
AUC (95% CI)	0.82 (0.71–0.92)
Optimal cutoff (Youden)	5.5 points
Sensitivity	77.4%
Specificity	86.4%
Positive predictive value	61.5%
Negative predictive value	93.1%
Brier score	0.114
AUC excluding valve-in-valve (*N* = 114) (95% CI)	0.91 (0.85–0.96)

#### 3.8.3 Calibration: score–outcome association

Univariable logistic regression of AVB occurrence on the raw D-PACE score yielded an intercept of –3.58 (SE 0.57; *p* < 0.0001) and a slope of 0.47 (SE 0.09; *p* < 0.0001), corresponding to an odds ratio of 1.59 per one-point increment (95% CI 1.33–1.90). In practical terms, each additional D-PACE point increases the odds of late high-grade AVB by 59%; a patient with a score of 8 faces approximately 41-fold higher odds than one with a score of 0 (1.59⁸ ≈ 41). The model predicts a risk of approximately 2.7% at a score of 0, rising to approximately 54% at score 8% and 65% at score 9, consistent with the observed event distribution across strata.

The apparent Brier score was 0.114, indicating that predicted probabilities deviated from actual outcomes by a mean squared error of approximately 11%, a figure comparable to well-validated cardiovascular risk models. The flattened logistic slope partly reflects the inclusion of 27 valve-in-valve patients classified as score 0 per the original algorithm; when these patients are removed (Section 3.8.6), both the OR per point and model discrimination improve. Because this is an external validation study—in which the seven D-PACE variable weights are fixed rather than re-estimated—the events-per-variable ratio that governs model development does not directly apply. Nonetheless, the modest total of 31 events limits the precision of stratum-specific rate estimates and calibration metrics, warranting confirmation in larger Latin-American cohorts.

Of note, the original D-PACE algorithm assigns patients to risk strata rather than providing individual predicted probabilities; the logistic model above therefore represents a local recalibration. The comparison of observed versus predicted rates in Section 3.8.4 should accordingly be interpreted as the agreement between a *de novo* model and the observed data, not as a test of the original algorithm's calibration. With only three risk strata the calibration plot (Figure 5) has limited resolution; calibration by individual score values was not feasible given the small event counts per score level.

**Figure 5:**
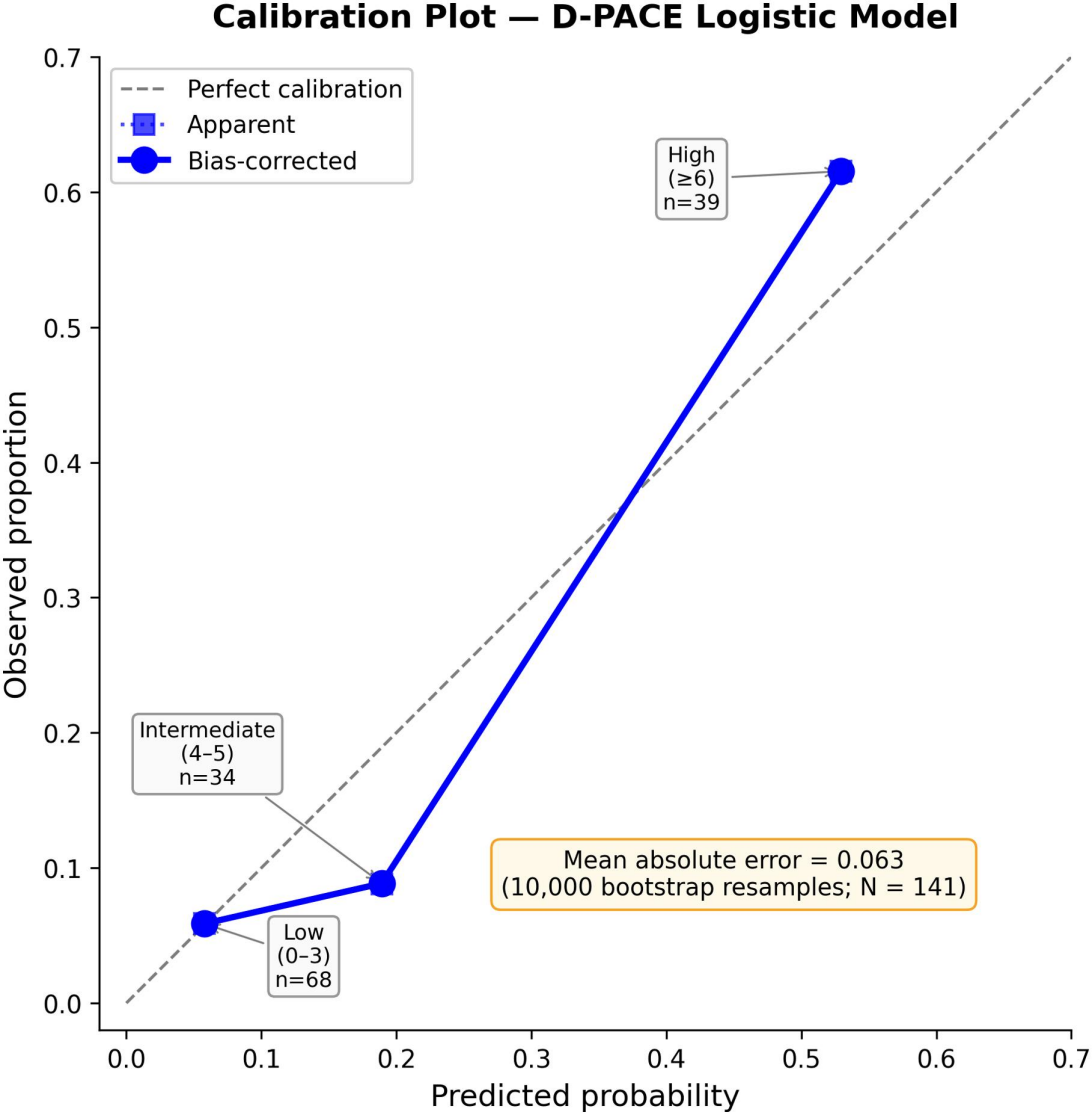
Bootstrap-corrected calibration plot of the logistic regression model (10,000 resamples; *N* = 141). The dotted line shows the apparent calibration, the solid line shows the bias-corrected estimate, and the dashed line represents perfect calibration. Mean absolute erro*r* = 0.063.

#### 3.8.4 Calibration: predicted vs. observed event rates

The observed event rates and mean logistic-model–predicted risk estimates by D-PACE risk stratum are presented in Table 7. In the low-risk group, the observed rate (5.9%) closely approximated the model prediction (5.8%), although this rate was driven entirely by the four valve-in-valve patients classified as low-risk per the original algorithm. In the high-risk group, the observed rate (61.5%) and the predicted estimate (52.9%) were in broad agreement, confirming the clinical utility of this stratum for identifying patients requiring intensive monitoring. The intermediate group showed the largest discrepancy, with an observed rate of 8.8% versus a predicted estimate of 18.9%. From a clinical standpoint, this overprediction constitutes a fail-safe behavior: the model errs toward recommending more monitoring rather than less preferable for a condition in which missed events carry serious consequences, including sudden death.

**Table 7:** Observed event rates and predicted risk estimates by D-PACE risk stratum.

Risk stratum	Score range	N	Observed late AVB rate (%)	Mean predicted risk (%)
Low	0–3	68	5.9	5.8
Intermediate	4–5	34	8.8	18.9
High	≥6	39	61.5	52.9

AVB, atrioventricular block.

#### 3.8.5 Clinical utility

Decision curve analysis demonstrated that a D-PACE-guided monitoring strategy provided greater net benefit than both default strategies (treat all and treat none) across threshold probabilities from approximately 8% to 60%, spanning the full clinically plausible range for escalated conduction surveillance (Figure 6). Because the original algorithm assigns risk strata rather than individual probabilities, the decision curve evaluates the locally recalibrated logistic model; net benefit should therefore be interpreted as the utility achievable with site-specific recalibration rather than as a property of the published score. At a representative threshold of 20%, the level at which a clinician would consider prolonged telemetry worthwhile given its associated costs, the net benefit was approximately 0.14, equivalent to correctly identifying 14 additional true-positive patients per 100 evaluated after accounting for the harm-weighted cost of false-positive interventions. Above approximately 8%, the D-PACE-guided strategy provided greater net benefit than treating all patients; the treat-all curve crossed zero near the cohort prevalence (∼22%), beyond which universal monitoring produced net harm, whereas D-PACE continued to provide positive net benefit up to approximately 60% by concentrating surveillance on higher-risk patients—indicating robust clinical value even in settings where the consequences of missed late AVB are weighted heavily, a consideration particularly relevant in resource-constrained environments. Bootstrap optimism correction (1,000 resamples) produced net benefit curves that closely overlapped with the apparent estimates, suggesting minimal overfitting.

**Figure 6:**
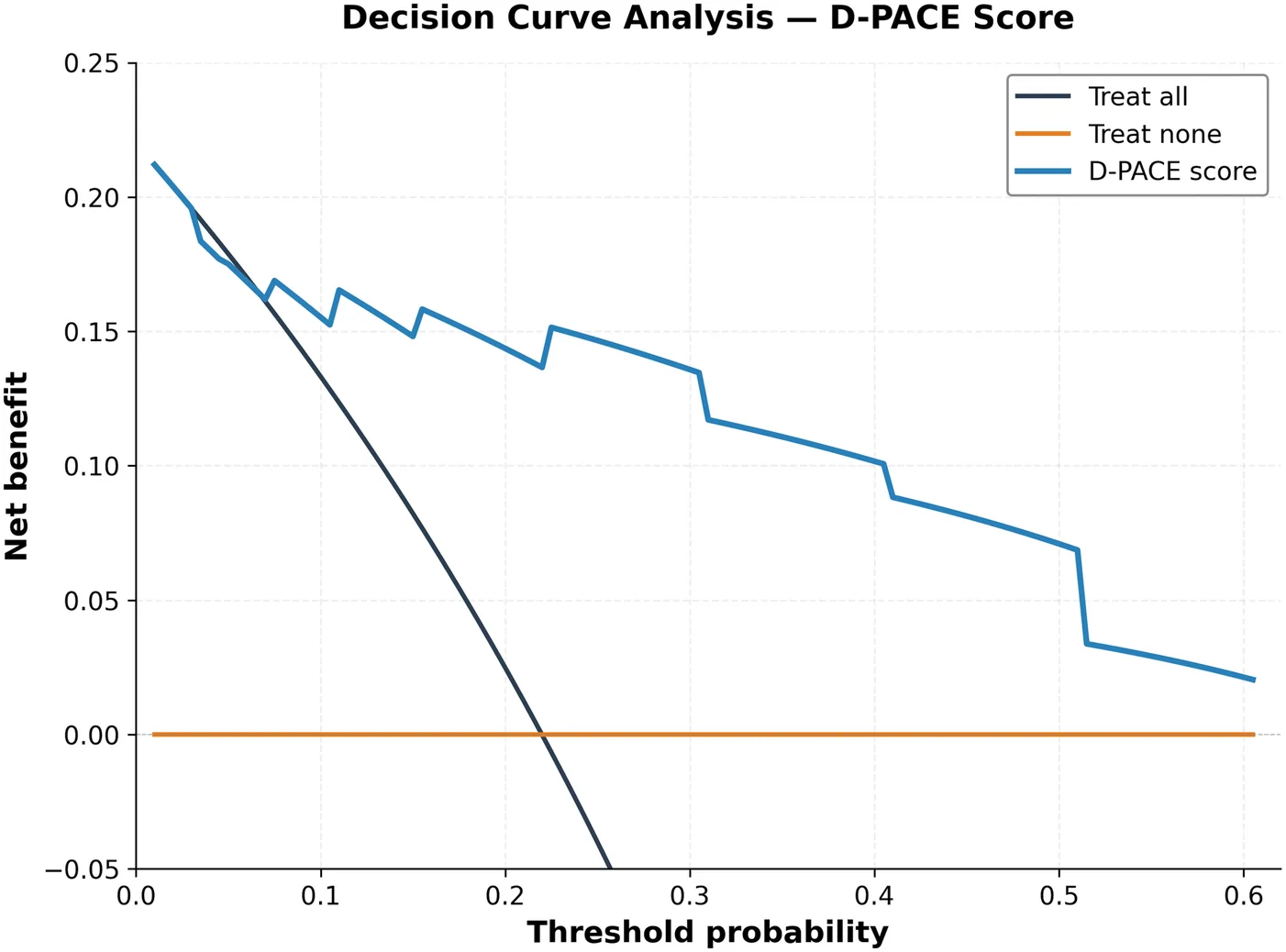
Decision curve analysis for the D-PACE score. Net benefit is plotted across threshold probabilities (0%–60%). The D-PACE-guided strategy (blue) provides greater net benefit than both the treat-all strategy (dark gray) and the treat-none strategy (orange) across threshold probabilities from approximately 8% to 60%, confirming the clinical utility of the score for guiding post-procedural conduction surveillance decisions.

#### 3.8.6 Sensitivity and exploratory analyses of the valve-in-valve subgroup

Because the original D-PACE algorithm classifies valve-in-valve patients as low-risk without applying the seven-variable scoring, a planned sensitivity analysis was performed after removing the 27 valve-in-valve cases. In the resulting cohort (*N* = 114; 27 late AVB events, 23.7%), the AUC rose to 0.91 (95% CI 0.85–0.96; percentile bootstrap). At the same cutoff of 5.5 points, sensitivity was 88.9%, specificity 82.8%, PPV 61.5%, and NPV 96.0%. The low-risk stratum (score 0–3; *N* = 41) contained zero late AVB events, compared with 5.9% in the full cohort—a difference entirely attributable to the four valve-in-valve patients who developed late AVB despite being classified as low-risk. Intermediate-risk (8.8%, 3/34) and high-risk (61.5%, 24/39) event rates were unchanged. These results indicate that the D-PACE seven-variable scoring achieves excellent discrimination in the population for which it was designed, and that the automatic low-risk classification of valve-in-valve patients—rather than any deficiency in the scoring algorithm—accounts for the lower AUC in the primary analysis.

In an exploratory *post hoc* analysis, the full seven-variable D-PACE scoring was applied to all 141 patients—including the 27 valve-in-valve cases, which were scored identically to non-valve-in-valve patients rather than receiving the automatic low-risk designation. Under this approach, the full-cohort AUC rose to 0.90 (95% CI 0.85–0.95; sensitivity 90.3%, specificity 79.1%, PPV 54.9%, NPV 96.7% at a cutoff of 5.5 points; OR 1.97 per point, 95% CI 1.55–2.49; Brier score 0.107). Because valve-in-valve patients now received computed scores rather than an automatic zero, the distribution across strata shifted accordingly: the low-risk stratum (score 0–3; *N* = 48, vs. 68 in the primary analysis) contained zero late AVB events, the intermediate stratum (score 4–5; *N* = 42, vs. 34) contained 3 events (7.1%), and the high-risk stratum (score ≥6; *N* = 51, vs. 39) captured 28 of 31 events (90.3%). Within the valve-in-valve subgroup alone (*N* = 27, 4 events), the seven-variable score yielded an AUC of 0.91 (95% CI 0.78–1.00): all four valve-in-valve patients who developed late AVB scored ≥7 (scores 7, 8, 10, 11), while the remaining 23 without AVB had a median score of 5 (IQR 3–6). The wide confidence interval and small event count preclude definitive conclusions, but these findings suggest that applying the standard seven-variable D-PACE scoring to valve-in-valve patients—rather than assigning them a blanket low-risk designation—may improve risk stratification in cohorts with heterogeneous underlying prostheses; this hypothesis warrants confirmation in larger samples.

#### 3.8.7 Temporal analysis

To assess score performance across evolving practice patterns, the cohort was divided into an early period (2012–2018; *N* = 42, 12 events) and a later period (2019–2025; *N* = 99, 19 events). Mean implant depth decreased from 6.7 ± 2.1 mm to 3.8 ± 2.0 mm, and the late AVB rate fell from 28.6% (95% CI 15.7–44.6) to 19.2% (95% CI 12.0–28.3). In the early period, the D-PACE AUC was 0.97 (95% CI 0.92–1.00), likely inflated by the small sample with wide score dispersion and no valve-in-valve events. In the later period, the AUC was 0.74 (95% CI 0.58–0.89) in the full cohort—reflecting the concentration of valve-in-valve patients in this era—and 0.90 (95% CI 0.82–0.96) after excluding valve-in-valve cases. These data suggest that discriminative performance remains robust in the contemporary-practice subset.

## 4 Discussion

This study provides the first external validation of the D-PACE score outside Europe and yields four key findings. First, applying the D-PACE algorithm exactly as published, with valve-in-valve patients assigned a score of 0, yielded good discrimination in the full cohort (AUC 0.82; 95% CI 0.71–0.92). Second, the seven-variable D-PACE scoring demonstrates excellent discrimination in non-valve-in-valve patients (AUC 0.91; 95% CI 0.85–0.96), numerically exceeding both the original European derivation (AUC 0.879) and external validation cohorts (AUC 0.799), although formal statistical comparison across independent cohorts was not performed. Third, the algorithm's automatic low-risk classification of valve-in-valve patients is not supported by our data: 4 of 27 such patients (14.8%) developed late AVB. Fourth, applying the full seven-variable scoring uniformly to all patients, including valve-in-valve cases, raised the full-cohort AUC to 0.90 (Section 3.8.6), highlighting the robustness of the scoring variables and the need to revisit the automatic low-risk classification rule. As noted in a related editorial (21), practical tools to guide early post-TAVR discharge decisions are a pressing clinical need. Our findings demonstrate that the electrocardiographic and procedural variables embedded in D-PACE retain their predictive capacity in a cohort with a distinctly different case-mix, device distribution, and learning-curve trajectory from the original European populations.

### 4.1 Comparison with the original D-PACE study

Although overall discrimination was comparable, absolute event rates differed meaningfully from the European experience (Table 8). The late high-grade AVB rate in our high-risk stratum (61.5%) was substantially higher than the 8.7–20.8% reported in the original study. Similarly, the low-risk group rate (5.9%) exceeded the <1% observed by Bendandi et al. (17), although this difference was entirely attributable to valve-in-valve patients: among non-valve-in-valve patients, the low-risk stratum contained zero events. Several interacting factors likely explain the high-risk stratum disparity.

**Table 8:** Comparison of D-PACE performance: original European cohorts vs. Colombian validation.

D-PACE stratum	Original study observed rate	Colombian cohort observed rate	Comment
Low (0–3)	<1% (derivation 0.45%; validation 0.64%)	5.9% (4/68)	Four events, all in valve-in-valve patients
Intermediate (4–5)	3.96% (derivation)/1.12% (validation)	8.8% (3/34)	Higher event rate than both European cohorts
High (≥6)	20.83% (derivation)/8.71% (validation)	61.5% (24/39)	Highest event concentration; supports intensive monitoring

First, self-expanding valve prevalence in our cohort (57.4%) exceeded that of the European derivation set (27.6%) but was lower than the external validation set (68.8%), indicating that valve type alone does not account for the higher event rates. Second, mean implant depth (4.7 mm overall, 6.2 mm in the AVB group) exceeded the <3 mm targets now standard with contemporary optimization techniques. Because implantation depth carries the highest maximum weight in the D-PACE algorithm, this variable contributes substantially to cumulative scores and, by extension, to the observed event rates in higher strata. A temporal analysis of our cohort (Section 3.8.7) documents progressive adoption of best practices: mean implant depth decreased from 6.7 ± 2.1 mm in the early period (2012–2018) to 3.8 ± 2.0 mm in the later period (2019–2025), paralleled by a reduction in the late AVB rate from 28.6% (12/42) to 19.2% (19/99)—a trajectory consistent with the learning-curve evolution reported by other programs. Third, the baseline prevalence of pre-existing RBBB (9.2%) and the high rate of new-onset persistent LBBB (28.4%) further compound cumulative D-PACE scores. The interaction of these three factors—rather than any single variable—most plausibly explains the elevated high-risk stratum event rates. This interpretation aligns with recent data from Massoullie et al. (22), who demonstrated that PR interval prolongation ≥40 ms at day 1 after TAVR is a strong predictor of high-grade AVB (hazard ratio 2.8), particularly in patients with QRS ≥100 ms—lending independent support to the PR-based components of the D-PACE algorithm.

The higher prevalence of bicuspid aortic valve morphology in our cohort (22.0% vs. the typical 5%–10% in European TAVR series) also warrants consideration. Bicuspid anatomy alters the prosthesis–outflow tract interaction, potentially modifying conduction injury risk; however, the late AVB rate was similar in bicuspid and tricuspid patients (25.8% vs. 20.9%), and mean implant depth was actually lower in the bicuspid subgroup (3.2 ± 1.4 vs. 5.1 ± 2.5 mm)—likely reflecting operator caution during valve sizing in asymmetric annuli. The elevated bicuspid prevalence therefore does not appear to drive the excess event rates but represents a distinctive feature of this Latin-American cohort that may warrant dedicated study.

Two additional methodological considerations merit discussion. The first is whether the events we detected were clinically meaningful or artifacts of intensive surveillance. Thirty of the 31 patients with late high-grade AVB (96.8%) underwent permanent pacemaker implantation on a contemporaneous clinical decision by the treating team, taken independently of and in most cases years before any retrospective D-PACE calculation; the single exception was a patient who died before implantation. Twenty-four of the 31 events (77.4%) were persistent and documented on a formal 12-lead electrocardiogram. The events were therefore clinically actionable rather than incidental findings of intensive monitoring. That said, our institution maintains continuous telemetry for a minimum of 72 h post-TAVR, a protocol that captures transient or paroxysmal episodes that shorter or intermittent monitoring would miss, and the seven transient events (22.6%) are consistent with a contribution of ascertainment intensity to the higher observed rate. Fournier et al. (23) recently demonstrated that nearly half of TAVR patients can be safely managed without continuous telemetry when stratified by conduction risk profile, which reinforces the value of D-PACE for identifying which patients genuinely require prolonged monitoring. Additionally, although identical definitions of high-grade AVB were applied across studies (≥Mobitz type II), subtle differences in adjudication—such as the classification of transient complete heart block episodes captured on telemetry but not on a formal ECG—cannot be fully excluded in a retrospective design.

The logistic model (univariable odds ratio 1.59 per D-PACE point; AUC 0.82, 95% CI 0.71–0.92) confirmed that despite higher absolute event rates, the relative ranking of risk was preserved. The AUC improved numerically when valve-in-valve cases were removed (0.91, 95% CI 0.85–0.96; see Sensitivity Analysis; Section 3.8.6), demonstrating excellent discrimination in the population for which the seven-variable scoring was designed, with robust performance maintained in the contemporary-practice subset (Section 3.8.7). The score differential between groups was driven predominantly by new-onset persistent LBBB, implantation depth, and next-day PR prolongation—two early post-procedural ECG signals amenable to timely detection and one operator-modifiable procedural variable.

### 4.2 Clinical implications of risk stratification

Decision curve analysis confirmed that D-PACE provides clinical utility beyond default strategies across the full range of plausible decision thresholds, supporting its integration into post-procedural discharge pathways. The NPV of 93.1% at the optimal cutoff (≥5.5 points) indicates that fewer than 1 in 14 patients classified as low-to-intermediate risk will develop late AVB. When valve-in-valve patients are excluded, the NPV rises to 96.0% with zero events in the low-risk stratum—a safety margin that meets the threshold required for confident early-discharge decision-making.

The clinical relevance of this risk stratification is underscored by the steep gradient of event rates across strata. Among non-valve-in-valve patients scoring 0–3 points, the AVB rate was 0%, supporting the safety of early discharge (24–48 h) with conventional ambulatory follow-up and serial ECG monitoring (13, 24, 25). In our cohort, the 41 non-valve-in-valve patients classified as low-risk represent 29% of the population; institutional TAVR volume has grown progressively over the study period, reaching approximately 25 procedures per year in recent years (2022–2024). The potential resource implication is best framed as an illustrative scenario rather than an estimate. If low-risk patients were discharged at 48 h instead of the observed mean of 6.3 days, and if annual volume and case-mix remained as in 2022–2024, the arithmetic yields on the order of 30 post-TAVR bed-days per year. This calculation assumes that early discharge is clinically appropriate in every low-risk patient, that no readmission offsets the saving, and that bed occupancy is the binding constraint; none of these assumptions was tested here, and the figure should be read as an order of magnitude rather than a projected saving. The safety of such abbreviated protocols is further supported by prospective data showing that selective telemetry strategies guided by conduction risk can be implemented without adverse events (23).

In the high-risk group (score ≥6), three of every five patients developed high-grade AVB within 30 days, justifying prolonged telemetry monitoring or, in selected cases, consideration of prophylactic pacemaker implantation prior to discharge (26, 27). The median onset of late AVB at day 2 further emphasizes that most delayed events occur within the first 72 h—a window amenable to in-hospital monitoring guided by D-PACE stratification. Salis et al. (28) validated the 2021 ESC guideline criteria for assessing pre-existing RBBB patients undergoing TAVR, confirming that a delta PR ≥20 ms and QRS axis change reliably predict conduction progression—findings consistent with the ECG-based variables embedded in D-PACE.

The intermediate-risk group (score 4–5) poses the most challenging clinical scenario, with an 8.8% event rate that precludes both unconditional early discharge and automatic prolonged monitoring. For these patients, individualized strategies—such as ambulatory cardiac rhythm monitoring during the first two weeks (29) or a lower threshold for PPI when concurrent anatomical risk factors are present—may be warranted. The multicenter ECG algorithm proposed by Badertscher et al. (30) for risk stratification in new-onset LBBB may offer complementary prognostic value, potentially refining decision-making in this intermediate group.

### 4.3 Pacemaker implantation rate

The 21.3% shown in Table 4 is a conditional rate: it is the proportion of the analytic cohort receiving a pacemaker, and that cohort by construction excludes patients with a prior device, patients paced for indications other than late high-grade AVB, and patients whose block occurred within the first 24 h. It is therefore not comparable with the overall post-TAVR implantation rates reported in registries. Among all 170 pacemaker-naive patients screened during the study period, 39 (22.9%) underwent permanent pacemaker implantation within 30 days for any indication, and 33 (19.4%) did so for high-grade AVB. Because the present analysis concerns delayed rather than early conduction disturbance, the more appropriate comparator is the late implantation rate of 7.5% beyond 48 h reported by CONDUCT-TAVI (14), against which our cohort shows a higher burden of delayed events, consistent with the predominance of self-expanding devices and the deeper implant positions characteristic of our program's early years. As described in Section 4.1, temporal analysis confirmed a progressive reduction in mean implant depth, consistent with the adoption of contemporary techniques including the cusp-overlap projection (31).

This trend illustrates a distinguishing feature of D-PACE: unlike most predictive models that rely exclusively on fixed patient characteristics, D-PACE incorporates implantation depth—an operator-modifiable variable carrying up to 4 points. The temporal analysis (Section 3.8.7) underscores this dynamic: the early-period AUC (0.97, 95% CI 0.92–1.00) is likely inflated by the small sample of 42 patients with wide score dispersion and no valve-in-valve events, whereas the later-period AUC (0.74, 95% CI 0.58–0.89, overall; 0.90, 95% CI 0.82–0.96, excluding valve-in-valve) illustrates the interplay between scoring accuracy and valve-in-valve classification. This dual nature—prognostic and potentially modifiable—suggests that D-PACE could serve not only to stratify risk *post hoc* but also to inform intraoperative depth targets that minimize conduction injury.

The valve-in-valve subgroup constitutes our most novel finding. In the original D-PACE study, none of 130 patients with conventional surgical bioprostheses developed late AVB, and the algorithm accordingly classifies all valve-in-valve patients as low-risk without applying the seven-variable scoring (17). In our cohort, however, 4 of 27 valve-in-valve patients (14.8%) developed late AVB requiring PPI—all with implant depths ≥6.9 mm and all presenting as third-degree AVB. This discrepancy likely reflects the heterogeneity of the underlying prostheses: the affected patients had St Jude Epic, Trifecta, and sutureless valves, which may lack the rigid ring geometry that conferred protection in the European cohort. As detailed in Section 3.8.6, removing the valve-in-valve subgroup raised the AUC from 0.82 to 0.91 and eliminated all low-risk events; applying the full seven-variable scoring classified all four as high-risk (scores 7–11), yielding a subgroup AUC of 0.91 (95% CI 0.78–1.00)—an estimate whose precision is limited by the small sample. Nonetheless, this observation suggests that applying the standard seven-variable scoring—rather than a blanket low-risk exemption—may improve risk stratification in cohorts with heterogeneous underlying prostheses, a hypothesis that warrants prospective confirmation.

### 4.4 Mortality and conduction disturbances

The striking difference in 30-day mortality between the AVB (12.9%) and no-AVB groups (0.9%) reinforces established associations between post-TAVR conduction disturbances and impaired survival (7, 10). Three of the four deaths in the AVB group (three of five deaths overall) were adjudicated as cardiovascular, consistent with, though not establishing, a mechanistic contribution. Several non-exclusive pathways may underlie this association: acute hemodynamic compromise during complete heart block before pacemaker implantation, ventricular dyssynchrony imposed by right ventricular apical pacing, and the higher periprocedural complexity inherent to cases with deeper implant positions (6, 32, 33). The one AVB patient who died before device implantation illustrates the narrow therapeutic window for managing complete heart block in elderly TAVR recipients.

Although the sample size precludes multivariable adjustment, the magnitude of the mortality difference (crude OR 16.1; 95% CI 1.7–150.4, exact method) parallels findings from the meta-analysis by Faroux et al. (10), in which post-TAVR conduction disturbances were independently associated with a 30% increase in long-term mortality. The wide confidence interval reflects the small number of events and precludes definitive causal inference, but the point estimate is consistent with a clinically important association. These observations argue that early identification of high-risk patients via D-PACE may afford not only logistical advantages but a shorter interval to definitive pacing; whether this translates into a survival benefit was not tested in this study and would require prospective evaluation.

### 4.5 Relevance for Latin-American TAVR programs

Latin-American TAVR programs face specific challenges—heterogeneous device availability, evolving operator experience, and healthcare resource constraints—that make prolonged post-procedural monitoring disproportionately costly. Our results demonstrate that D-PACE, originally developed in high-volume European centers, translates effectively to this context. Systematic integration into discharge pathways could (a) shorten hospital stays for the 41 non-valve-in-valve patients classified as low-risk (29% of the cohort), freeing intermediate care unit beds; (b) concentrate monitoring resources on the 27.7% scoring ≥6, among whom the majority of events occur; and (c) provide the first evidence-based framework for risk-stratified post-TAVR discharge in the region—a tool currently absent from Latin-American cardiovascular guidelines. The finding that valve-in-valve patients classified as low-risk by the original algorithm had a 14.8% AVB rate warrants particular attention in programs with a substantial valve-in-valve caseload.

### 4.6 Limitations

Several limitations should be acknowledged. First, the single-center, retrospective design introduces potential selection and information biases; the sample size of 141 patients (31 events) limits the precision of stratum-specific rate estimates and subgroup analyses. Second, the heterogeneity of valve generations and models over the 13-year study period introduces confounding related to evolving device technology, operator experience, and implantation technique that cannot be disentangled from device-era effects. Third, D-PACE requires measurable PR intervals, restricting its application to patients in sinus rhythm; the exclusion of 5 patients with atrial fibrillation (2.9% of those screened) limits generalizability to the approximately 85%–90% of TAVR candidates in sinus rhythm, and 5 additional patients were excluded for incomplete data or loss to follow-up. Fourth, differences in telemetry duration between our institution and the European centers may contribute to the higher detected event rates, as more intensive surveillance captures a greater proportion of transient or paroxysmal episodes. Fifth, exclusion of patients who died within 30 days without AVB introduces competing-risk bias that may underestimate the true incidence of late AVB; dedicated competing-risk regression (Fine–Gray) was not performed given the low mortality rate (5 deaths), but this should be considered in larger multicenter studies. Sixth, partial incorporation bias cannot be excluded: D-PACE relies on post-procedural ECG variables (new-onset LBBB, PR interval change) that may share pathophysiological mechanisms with the outcome (high-grade AVB), potentially inflating apparent discrimination; moreover, because both score calculation and outcome adjudication were performed retrospectively by overlapping investigators, formal blinding of the outcome assessment to the D-PACE score was not enforced, although the standardized ECG and telemetry criteria minimize subjective judgement. Finally, external validation in multicenter Latin-American cohorts with contemporary-generation devices is needed before these findings can be generalized across the region.

## 5 Conclusions

In this consecutive Colombian cohort of 141 TAVR patients, the D-PACE score applied exactly as published showed good discrimination for late high-grade atrioventricular block (AUC 0.82, 95% CI 0.71–0.92), with clinical utility across decision thresholds. In the sensitivity analysis restricted to non-valve-in-valve patients, discrimination was excellent (AUC 0.91, 95% CI 0.85–0.96). The algorithm's automatic low-risk classification of valve-in-valve patients was not supported (14.8% AVB rate); applying the full seven-variable scoring uniformly raised the cohort AUC to 0.90 and correctly identified all four valve-in-valve AVB cases—a finding that warrants prospective confirmation in larger samples. Risk-stratified management appears feasible: the low-risk category contained zero events among non-valve-in-valve patients, supporting early discharge, whereas the high-risk category warrants prolonged telemetry. These findings validate the D-PACE scoring variables in a Latin-American population with a distinctly different case-mix from the original European cohorts.

## Data Availability

The raw data supporting the conclusions of this article will be made available by the authors, without undue reservation.
