# Supplementary material for: External validation of the D-PACE score for predicting late high-grade atrioventricular block after transcatheter aortic valve replacement in a Colombian cohort
Source: Front Cardiovasc Med. 2026 Oct 14;13:1905818. doi: 10.3389/fcvm.2026.1905818 (PMC13713820; doi:10.3389/fcvm.2026.1905818)
Supplement: Supplementary file 1 [file Table1.docx]

**Supplementary Table S1. STROBE Checklist for Cohort Studies**

**Study:** External Validation of the D-PACE Score for Predicting Late High-Grade Atrioventricular Block After Transcatheter Aortic Valve Replacement in a Colombian Cohort

| **Item No.** | **STROBE Item** | **Recommendation** | **Reported on Section / Table / Figure** |
| --- | --- | --- | --- |
| **Title and abstract** |  |  |  |
| 1a | Title | Indicate the study's design with a commonly used term in the title or the abstract | Title ("External Validation … in a Colombian Cohort"); Abstract ("retrospective cohort") |
| 1b | Abstract | Provide an informative and balanced summary of what was done and what was found | Abstract |
| **Introduction** |  |  |  |
| 2 | Background/rationale | Explain the scientific background and rationale for the investigation being reported | Section 1 (Introduction), paragraphs 1–5 |
| 3 | Objectives | State specific objectives, including any pre-specified hypotheses | Section 1, final paragraph |
| **Methods** |  |  |  |
| 4 | Study design | Present key elements of study design early in the paper | Section 2.1 ("single-center, retrospective cohort study") |
| 5 | Setting | Describe the setting, locations, and relevant dates, including periods of recruitment, exposure, follow-up, and data collection | Section 2.1 (Fundación Clínica Shaio, Bogotá; January 2012 – March 2025) |
| 6a | Participants | Give the eligibility criteria, and the sources and methods of selection of participants. Describe methods of follow-up | Sections 2.2 (inclusion/exclusion criteria), 2.3 (data collection) |
| 6b | Participants | For matched studies, give matching criteria and number of exposed and unexposed | Not applicable (unmatched cohort) |
| 7 | Variables | Clearly define all outcomes, exposures, predictors, potential confounders, and effect modifiers. Give diagnostic criteria, if applicable | Sections 2.3 (collected variables), 2.4 (D-PACE variables), 2.5 (outcomes) |
| 8 | Data sources / measurement | For each variable of interest, give sources of data and details of methods of assessment (measurement). Describe comparability of assessment methods if there is more than one group | Section 2.3 (electronic health records, catheterization databases, ECG registries; three ECG time points), Section 2.4 (implant depth measurement by two operators) |
| 9 | Bias | Describe any efforts to address potential sources of bias | Section 2.4 (independent score calculation by two investigators, consensus adjudication); Section 4.6, limitations 1, 4, 5, 8, 9 |
| 10 | Study size | Explain how the study size was arrived at | Section 3.1 (175 screened → 141 included); Figure 1 |
| 11 | Quantitative variables | Explain how quantitative variables were handled in the analyses. If applicable, describe which groupings were chosen and why | Section 2.6 (continuous vs. categorical presentation; Shapiro-Wilk for distribution; D-PACE as continuous predictor in logistic regression and as three risk strata) |
| 12a | Statistical methods | Describe all statistical methods, including those used to control for confounding | Section 2.6 (ROC/AUC with DeLong CIs, logistic regression, DCA with bootstrap, Clopper–Pearson CIs) |
| 12b | Statistical methods | Describe any methods used to examine subgroups and interactions | Section 2.6 (planned sensitivity analysis excluding valve-in-valve; exploratory post hoc ViV-scored analysis; temporal analysis) |
| 12c | Statistical methods | Explain how missing data were addressed | Section 2.2 (patients with incomplete data excluded); Section 3.8.1 ("all 31 with late AVB had complete data") |
| 12d | Statistical methods | If applicable, explain how loss to follow-up was addressed | Section 2.2 (excluded for incomplete follow-up); Figure 1 (n = 5 incomplete/lost) |
| 12e | Statistical methods | Describe any sensitivity analyses | Section 2.6 (sensitivity analysis excluding ViV; exploratory ViV-scored analysis; temporal analysis) |
| **Results** |  |  |  |
| 13a | Participants | Report numbers of individuals at each stage of study — e.g., numbers potentially eligible, examined for eligibility, confirmed eligible, included in the study, completing follow-up, and analysed | Section 3.1; Figure 1 (175 → 141) |
| 13b | Participants | Give reasons for non-participation at each stage | Figure 1 (AF n = 5, incomplete n = 5, death without AVB n = 9, prior PPM n = 5, non-AVB PPI n = 6, early AVB n = 4) |
| 13c | Participants | Consider use of a flow diagram | Figure 1 |
| 14a | Descriptive data | Give characteristics of study participants and information on exposures and potential confounders | Table 1 (baseline); Table 2 (procedural); Table 3 (ECG); Section 3.3 (echocardiographic/CTA) |
| 14b | Descriptive data | Indicate number of participants with missing data for each variable of interest | Section 3.3 (CT performed in 97.2%); Section 3.8.1 (complete D-PACE data in all 31 AVB patients) |
| 14c | Descriptive data | Summarise follow-up time | Section 2.5 (30-day follow-up); Section 3.7 (30-day outcomes) |
| 15 | Outcome data | Report numbers of outcome events or summary measures over time | Section 3.6 (31/141 late AVB; temporal distribution Figure 5); Section 3.7 (mortality, PPI, length of stay) |
| 16a | Main results | Give unadjusted estimates and, if applicable, confounder-adjusted estimates and their precision | Section 3.8.2 (AUC 0.82, 95% CI; sensitivity/specificity); Section 3.8.3 (OR 1.56, 95% CI 1.31–1.85); Table 5 (stratum-specific rates with 95% CIs) |
| 16b | Main results | Report category boundaries when continuous variables were categorized | Section 3.8.1 (low 0–3, intermediate 4–5, high ≥6); Section 2.4 (implant depth and PR interval categories) |
| 16c | Main results | If relevant, consider translating estimates of relative risk into absolute risk for a meaningful time period | Section 3.8.3 (absolute predicted risk at scores 0, 8, 9); Table 7 (observed vs. predicted absolute rates per stratum) |
| 17 | Other analyses | Report other analyses done — e.g., analyses of subgroups and interactions, and sensitivity analyses | Sections 3.8.6 (excl. ViV: AUC 0.90; ViV-scored: AUC 0.89; ViV subgroup: AUC 0.91), 3.8.7 (temporal analysis) |
| **Discussion** |  |  |  |
| 18 | Key results | Summarise key results with reference to study objectives | Section 4, opening paragraph (three key findings) |
| 19 | Limitations | Discuss limitations of the study, taking into account sources of potential bias or imprecision | Section 4.6 (11 limitations: First through Tenth + Finally) |
| 20 | Interpretation | Give a cautious overall interpretation of results considering objectives, limitations, multiplicity of analyses, results from similar studies, and other relevant evidence | Sections 4.1–4.5 |
| 21 | Generalisability | Discuss the generalisability (external validity) of the study results | Section 4.5 (Relevance for Latin-American TAVR Programs); Section 4.6, limitation 11 |
| **Other information** |  |  |  |
| 22 | Funding | Give the source of funding and the role of the funders for the present study and, if applicable, for the original study on which the present article is based | Funding section ("no specific grant") |

Reference: von Elm E, Altman DG, Egger M, Pocock SJ, Gøtzsche PC, Vandenbroucke JP; STROBE Initiative. The Strengthening the Reporting of Observational Studies in Epidemiology (STROBE) statement: guidelines for reporting observational studies. *Lancet.* 2007;370(9596):1453-1457.
