# Supplementary material for: External validation of the D-PACE score for predicting late high-grade atrioventricular block after transcatheter aortic valve replacement in a Colombian cohort
Source: Front Cardiovasc Med. 2026 Oct 14;13:1905818. doi: 10.3389/fcvm.2026.1905818 (PMC13713820; doi:10.3389/fcvm.2026.1905818)
Supplement: Supplementary file 2 [file Table2.docx]

**Supplementary Table S2. TRIPOD Checklist for External Validation Studies (Type 4)**

**Study:** External Validation of the D-PACE Score for Predicting Late High-Grade Atrioventricular Block After Transcatheter Aortic Valve Replacement in a Colombian Cohort

| **Item No.** | **TRIPOD Item** | **Checklist Item** | **Reported on Section / Table / Figure** |
| --- | --- | --- | --- |
| **Title and abstract** |  |  |  |
| 1 | Title | Identify the study as developing and/or validating a multivariable prediction model, the target population, and the outcome to be predicted | Title ("External Validation of the D-PACE Score for Predicting Late High-Grade Atrioventricular Block After Transcatheter Aortic Valve Replacement in a Colombian Cohort") |
| 2 | Abstract | Provide a summary of objectives, study design, setting, participants, sample size, predictors, outcome, statistical analysis, results, and conclusions | Abstract (structured: Background, Methods, Results, Conclusions) |
| **Introduction** |  |  |  |
| 3a | Background | Explain the medical context and rationale for developing or validating the multivariable prediction model, including references to existing models | Section 1, paragraphs 1–4 (clinical context of late AVB after TAVR; existing prediction tools including Janiszewski, CONDUCT-TAVI, D-PACE) |
| 3b | Objectives | Specify the objectives, including whether the study describes the development or validation of the model or both | Section 1, final paragraph ("externally validate the D-PACE score in a consecutive Colombian TAVR cohort and to characterize the clinical, procedural, and electrophysiological profile") |
| **Methods** |  |  |  |
| 4a | Source of data | Describe the study design or source of data (e.g., randomized trial, cohort, or registry data), separately for the development and validation data sets, if applicable | Section 2.1 ("single-center, retrospective cohort study"); original model: Bendandi et al. (2024) multicenter European derivation/validation |
| 4b | Source of data | Specify the key study dates, including start of accrual; end of accrual; and, if applicable, end of follow-up | Section 2.1 (January 1, 2012 – March 31, 2025; 30-day follow-up) |
| 5a | Participants | Specify key elements of the study setting (e.g., primary care, secondary care, general population) including number and location of centres | Section 2.1 (Fundación Clínica Shaio, tertiary cardiovascular center, Bogotá, Colombia; single center) |
| 5b | Participants | Describe eligibility criteria for participants | Section 2.2 (inclusion and exclusion criteria) |
| 5c | Participants | Give details of treatments received, if relevant | Section 3.4 (TAVR procedural details: valve types, implant depth, pre/post-dilation); Table 2 |
| 6a | Outcome | Clearly define the outcome that is predicted by the prediction model, including how and when assessed | Section 2.5 ("late high-grade AVB, defined as ≥Mobitz type II occurring between 24 hours and 30 days post-TAVR, confirmed by ECG or continuous telemetry") |
| 6b | Outcome | Report any actions to blind assessment of the outcome to be predicted | Section 4.6, limitation 9 (formal blinding not enforced; standardized ECG/telemetry criteria minimize subjectivity) |
| 7a | Predictors | Clearly define all predictors used in developing or validating the multivariable prediction model, including how and when they were measured | Section 2.4 (seven D-PACE variables with exact definitions, measurement timing, and scoring thresholds) |
| 7b | Predictors | Report any actions to blind assessment of predictors for the outcome and other predictors | Section 2.4 (scores independently calculated by two investigators; discrepancies resolved by consensus with third investigator) |
| 8 | Sample size | Explain how the study size was arrived at | Section 3.1 (175 screened, 34 excluded, 141 analyzed); Figure 1; Section 3.8.3 (discussion of 31 events relative to model validation requirements) |
| 9 | Missing data | Describe how missing data were handled (e.g., complete-case analysis, single imputation, multiple imputation) with details of any imputation method | Section 2.2 (patients with incomplete data excluded — complete-case analysis); Section 3.8.1 ("all 31 with late AVB had complete data for the seven D-PACE variables") |
| 10a | Statistical analysis methods | Describe how predictors were handled in the analyses | Section 2.4 (D-PACE computed per original algorithm: fixed weights, categorical thresholds for continuous predictors) |
| 10b | Statistical analysis methods | Specify type of model, all model-building procedures (including any predictor selection), and method for internal validation | Not applicable — external validation study; original model by Bendandi et al. (2024) referenced |
| 10c | Statistical analysis methods | For validation, describe how the predictions were calculated | Section 2.4 (seven-variable scoring per original publication; valve-in-valve patients classified as score 0 per original algorithm); Section 2.6 (local logistic recalibration) |
| 10d | Statistical analysis methods | Specify all measures used to assess model performance and, if relevant, to compare multiple models | Section 2.6 (AUC with DeLong 95% CI; sensitivity, specificity, PPV, NPV at Youden cutoff; logistic regression intercept, slope, OR per point; Brier score; DCA with bootstrap optimism correction; Clopper–Pearson CIs for stratum rates) |
| 10e | Statistical analysis methods | Describe any model updating (e.g., recalibration) arising from the validation, if done | Section 2.6 (local logistic regression as site-specific recalibration); Section 3.8.3 (recalibration results) |
| 11 | Risk groups | Provide details on how risk groups were created, if done | Section 2.4 (low 0–3, intermediate 4–5, high ≥6 — per original D-PACE strata) |
| 12 | Development vs. validation | For validation, identify any differences from the development data in setting, eligibility criteria, outcome, and predictors | Section 4.1 (Table 8: European vs. Colombian rates; differences in valve-type distribution, implant depth, LBBB rates, bicuspid prevalence, telemetry duration, ViV caseload) |
| **Results** |  |  |  |
| 13a | Participants | Describe the flow of participants through the study, including the number of participants with and without the outcome and, if applicable, a summary of the follow-up time. A diagram may be helpful | Figure 1 (flow diagram); Section 3.1 (141 analyzed: 31 AVB, 110 no AVB) |
| 13b | Participants | Describe the characteristics of the participants (including demographics, clinical features, available predictors), including the number of participants with missing data for predictors and outcome | Tables 1–3 (demographics, procedural, ECG); Section 3.3 (echocardiographic/CTA); Section 3.8.1 (complete predictor data) |
| 13c | Participants | For validation, show a comparison with the development data of the distribution of important variables (demographics, predictors and outcome) | Section 4.1, Table 8 (stratum-specific rates: original vs. Colombian); Section 4.1 text (valve type, implant depth, RBBB, LBBB comparisons) |
| 14a | Model performance | Report performance measures (with CIs) for the prediction model | Section 3.8.2, Table 6 (AUC 0.82, 95% CI 0.71–0.92; Se 77.4%, Sp 85.5%, PPV 60.0%, NPV 93.1%; Brier 0.119); Section 3.8.6 (excl. ViV AUC 0.90, 95% CI 0.85–0.96; ViV-scored AUC 0.89, 95% CI 0.84–0.95) |
| 14b | Model performance | If done, report the results from any model updating (i.e., model specification, model performance, recalibration) | Section 3.8.3 (logistic recalibration: intercept –3.46, slope 0.44, OR 1.56); Section 3.8.4, Table 7 (predicted vs. observed rates); Figure 3 (calibration plot) |
| **Discussion** |  |  |  |
| 15a | Interpretation | For validation, discuss the results with reference to performance in the development data, and any other validation data | Section 4.1 (comparison with European derivation AUC 0.879 and validation AUC 0.799; Table 8) |
| 15b | Interpretation | Give an overall interpretation of the results, considering objectives, limitations, results from similar studies, and other relevant evidence | Sections 4.1–4.5; Section 5 (Conclusions) |
| 16 | Limitations | Discuss any limitations of the study (such as nonrepresentative sample, few events per predictor, missing data) | Section 4.6 (11 limitations including sample size, heterogeneity, sinus rhythm restriction, telemetry differences, inter-observer agreement, missing membranous septum data, no EPS, competing risk, incorporation bias, no cost-effectiveness, need for multicenter validation) |
| 17 | Implications | For validation, discuss the results with reference to possible clinical use and the potential to improve the existing model | Sections 4.2 (clinical implications for discharge pathways), 4.3 (PPI rate and modifiable depth), 4.5 (Latin-American relevance); Section 5 (Conclusions) |
| **Other information** |  |  |  |
| 18 | Supplementary information | Provide information about the availability of supplementary resources, such as study protocol, Web calculator, and data sets | Data Availability Statement ("upon reasonable request") |
| 19a | Funding | Give the source of funding and the role of the funders for the present study | Funding section ("no specific grant") |
| 19b | Funding | For validation, give reference to or provide sufficient information about the development model for other investigators to validate the model | Section 2.4 (full D-PACE algorithm described); Reference: Bendandi et al. (2024) |

Reference: Collins GS, Reitsma JB, Altman DG, Moons KGM. Transparent Reporting of a multivariable prediction model for Individual Prognosis or Diagnosis (TRIPOD): the TRIPOD statement. *BMJ.* 2015;350:g7594.
